# Physical Activity, Sedentary Behavior, and Cardiorespiratory Fitness in Hazardous and Non-Hazardous Alcohol Consumers

**DOI:** 10.1177/0890117120985830

**Published:** 2021-01-07

**Authors:** Mats Hallgren, Davy Vancampfort, Thi-Thuy-Dung Nguyen, Elin Ekblom-Bak, Peter Wallin, Gunnar Andersson, Andreas Lundin

**Affiliations:** 1 Epidemiology of Psychiatric Conditions, Substance Use and Social Environment (EPiCSS), Department of Public Health Sciences, 27106 Karolinska Institutet, Stockholm, Sweden; 2 Department of Rehabilitation Sciences, University of Leuven; and University Psychiatric Center, Katholieke Universiteit Leuven, Belgium; 3 Medical Epidemiology and Biostatistics (MEB), 27106 Karolinska Institutet, Stockholm, Sweden; 4 Astrand Laboratory of Work Physiology, 42750 The Swedish School of Sport and Health Sciences, Stockholm, Sweden; 5 Research Department, HPI Health Profile Institute, Danderyd, Sweden

**Keywords:** alcohol, exercise, physical activity, fitness, sedentary behavior

## Abstract

**Purpose:**

To describe physical activity habits, sedentary behavior, and cardiorespiratory fitness levels among alcohol abstainers, hazardous and non-hazardous drinkers.

**Design:**

Cross-sectional study with data collected between 2017-19.

**Setting:**

Sweden.

**Subjects:**

Adults aged 18-65 years (n = 47,559; 59.4% male).

**Measures:**

During a routine health assessment, participants answered validated single-item questions regarding: habitual physical activity, structured exercise, and the percentage of time spent sedentary during leisure-time (past 30 days), and completed a 6-minute cycle ergometer test (V02max) to determine cardiorespiratory fitness (CRF). Participants were categorized as alcohol abstainers, non-hazardous drinkers or hazardous drinkers (low/high) based on the Alcohol Use Disorders Identification Test (AUDIT-C) cut-points for men and women.

**Analysis:**

Logistic regression models stratified by sex and age.

**Results:**

Compared to non-hazardous drinkers, the heaviest drinkers were less physically active (males: OR = 1.38, CI = 1.13-1.67, p = .001; females: OR = 1.41, CI = 1.01-1.97, p = .040) and more sedentary during leisure time (males: OR = 1.94, CI = 1.62-2.32, p = .000; females: OR = 1.62, CI = 1.21-2.16, p = .001). Apart from young females, the heaviest drinkers also did less structured exercise than non-hazardous drinkers (males: OR = 1.22, CI = 1.15-1.51, p = .000; females: OR = 1.43, CI = 1.15-1.78, p = .001). The strongest associations were seen among adults aged 40-65 years (shown here). High-hazardous drinking was associated with low CRF among older males only (OR = 1.19, CI = 1.00-1.41).

**Conclusion:**

Middle-aged adults with AUDIT-C scores of ≥6 (women) and ≥7 (men) were less physically active and more sedentary during leisure time and may be appropriate targets for physical activity interventions.

## Purpose

People with alcohol use disorder (AUD) experience an excess mortality rate 2-times higher than those without AUD.^1^ The prevalence of type-2 diabetes mellitus and the metabolic syndrome is higher in AUD compared to the general population, and cardiovascular deaths are twice as common.^1^ Related to these somatic conditions, depression and anxiety is also more prevalent in those with AUD.^2^

Regardless of drinking status, physical inactivity—that is, achieving less than the recommended 150 minutes/week of moderate-to-vigorous physical activity—is shown to increase the risk of poor somatic and psychiatric health.^3^ In a series of studies involving inpatients treated for AUD, Vancampfort and colleagues showed that physical activity levels and cardiorespiratory fitness (CRF) were significantly lower than age-gender matched healthy controls.^4^ Functional exercise capacity was also shown to be impaired and detrimentally associated with global functioning.^5^ These studies highlight the potential importance of physical activity in the treatment of AUD and related health problems. Several trials have evaluated the effects of structured exercise interventions for AUD.^6^ Systematic reviews indicate that exercise-based interventions have positive effects on depression, life quality and somatic health indicators, but effects on alcohol consumption are less certain.^7,8^

The onset of AUD is often preceded by a period of increasingly frequent and/or heavy alcohol consumption. “Hazardous drinking” has been defined as a quantity or pattern of alcohol use that places someone at risk for adverse health events.^9^ While there is no universally agreed definition, cut-points for hazardous drinking have been established using the Alcohol Use Disorders Identification Test (AUDIT). In Sweden, scores of ≥5 (men) and ≥4 (women) on AUDIT-C (the first 3 AUDIT questionnaire items) are generally accepted as thresholds for hazardous drinking and shown to correlate highly with AUDIT total scores.^10,11^ The prevalence of hazardous drinking varies between countries depending on the definition used. Recent estimates suggest that between 15-20% of Swedish adults drink at hazardous levels (past 12 months).^12^ Worldwide, hazardous drinking is more prevalent among men and younger adults. A US study reported the 12-month prevalence of “risky” drinking was 30% among 18-39 year olds.^13^ A recognized challenge is that while hazardous drinkers contribute substantially to alcohol-related deaths, injuries, and social problems,^14^ they rarely seek professional support for their drinking habits.^15^ Non-stigmatizing interventions that promote a physically active lifestyle have potential to increase help-seeking and improve somatic and psychiatric wellbeing.^16^

Although physical activity and it’s subset, structured exercise, is increasingly used to treat substance use disorders,^8^ general population studies describing the full spectrum of physical activity habits among hazardous and non-hazardous drinkers remain absent. These epidemiological data could inform the design of clinical trials and research-driven prevention strategies. In particular, descriptions of sedentary behavior (too much sitting, as opposed to too little exercise),^17^ and cardiorespiratory fitness (CRF) are needed. Adults in high-income countries are sedentary for ∼8–12 hours/day,^18^ Higher volumes of sedentary time have been linked to greater risk of cardiovascular disease and premature mortality,^19^ and these associations also remain after adjustment for moderate-to-vigorous physical activity.^20^ Similarly, CRF is shown to reduce the risk of multiple non-communicable diseases, including common mental health problems.^21^ Currently, there are no studies comparing levels of sedentary behavior and CRF in adults based on their drinking status.

We examined physical activity habits (including habitual physical activity, structured exercise, and sedentary behavior), and CRF levels, among alcohol abstainers, non-hazardous and hazardous drinkers. As sex and age are known moderators of alcohol consumption,^22^ we stratified all analyses based on these 2 participant characteristics.

## Method

We referred to the Strengthening the Reporting of Observational studies in Epidemiology (STROBE) guidelines while conducting this study.

### Study Population

Data originate from the Swedish Health Profile Assessment (HPA) database (www.hpihealth.se/). HPA includes a 1-page questionnaire about lifestyle and health experiences, measurement of anthropometrics, and estimation of VO_2max_ from a submaximal fitness test on a cycle ergometer. The HPA is offered nationally to all employees working for organizations connected to occupational or health related services (OHS), covering an estimated 72% of all employees.^23^ Participation is voluntary and free-of-charge. Although HPA has been running since 1976, we will base our analyses on data collected from January 2017 (when questions on relevant variables were first introduced) to June 2019. The total initial sample comprised 48,287 participants, of which 47,559 (98.5%) had data on alcohol consumption (analytic sample). The original study complies with the guidelines of the Declaration of Helsinki. The Research Ethics Vetting Board in Stockholm approved the original study (Dnr 2015/1864-31/2 and 2016/9-32). Informed consent was obtained from participants after the procedure was fully explained.

### Measures

***Alcohol consumption:*** This was assessed using the first 3 items of the AUDIT questionnaire, AUDIT-C (item 1 = frequency; item 2 = quantity, expressed as standard drinks; item 3 = frequency of heavy episodic drinking).^24^ All questions referred to the past 6 months. Items are scored from 0 to 4 and summed. Hazardous drinkers were defined as those scoring ≥4 (women) and ≥5 (men) on AUDIT-C. As the proportion of hazardous drinkers in the study population was large (33.3%), and to assess potential differences within this category based on AUDIT-C scores, we divided hazardous drinkers into those scoring 5-6 points and ≥7 AUDIT-C points (men), and those scoring 4-5 points and ≥6 AUDIT-C points (women), respectively. Cut-points were determined based on the distribution of scores, with the aim of retaining a sufficient number of participants in each group after the data were stratified. We refer to these 2 categories as “low-hazardous” and “high-hazardous” drinkers. For both sexes, alcohol abstainers were defined as those scoring 0 on AUDIT-C.

#### Physical activity variables

***Exercise frequency:*** This was assessed with the question: During the past 30 days…“I exercise/train…” with 8 response alternatives; Never, Sometimes, 1 time/week, 2 times/week, 3 times/week, 4 times/week, 5 times/week, ≥6 times/week. We coded responses to the first 2 categories (Never, Sometimes) as 1 = low exercise; all other responses were coded as 0. To estimate the average (mean) number of weekly exercise sessions, a continuous variable was created by converting these responses into numbers (where Sometimes = 0.5, and ≥6 times/week = 6).

***Cardiorespiratory fitness:*** This was assessed as VO_2max_, (maximal oxygen uptake) expressed as ml/min/kg, estimated from heart rate response after completing the Åstrand 6-minute submaximal exercise test on a Monark cycle ergometer.^25^ We calculated the mean VO_2max_, test score. As recommended,^26^ values ≤32 ml/min/kg were coded as 1 = low CRF. Scores >32 ml/min/kg were coded as 0.

***Leisure-time sedentary behavior:*** This was assessed with the question “I sit still during my leisure-time…” with 5 response alternatives: Almost always, 75% of the time, 50% of the time, 25% of the time, Almost never. The first 2 responses were coded as 1 = high sedentary; all other responses were coded as 0.

***Habitual physical activity:*** This was assessed with the question: “Besides exercise, I choose physical activities…I.e. walking, cycling, gardening etc…” with 5 response alternatives: Never, 1 day/week, Several days/week, Every day, Many times/day. The first 2 categories were coded as 1 = low physical activity (PA); all other responses were coded as 0. To estimate the mean PA sessions per week, a continuous variable was created by converting the response alternatives as follows: Never = 0, 1 day/week = 1, Several days/week = 3.5, Every day = 7, Many times/day = 14 (where “many” was conservatively interpreted as twice a day).

The physical activity variables were categorized based on face validity (i.e. logical cut-points given our research aims). The distribution of data was also considered, with the goal of retaining sufficient numbers for analysis after stratification.

#### Other descriptive variables

***Relationship status:*** This was assessed by asking participants their current relationship status. Responses were coded as: None, (yes but…) Living apart, and (yes…) Living together.

***Education level:*** This was not directly assessed in the HPA survey, but derived by converting occupation codes from the Swedish Standard Classification of Occupation (2012)/International Standard Classification of Occupation (2008) into 4 education levels: Primary school, Secondary/ tertiary (2 years), Vocational tertiary (3-4 years), Theoretical tertiary ≥ 3 years. The conversion process is described elsewhere.^27^

***Body mass index (BMI):*** Weight was assessed with a calibrated scale in lightweight clothing to the nearest 0.5 kg. Height was measured to the nearest 0.5 cm using a wall-mounted stadiometer. With these 2 variables, body mass index (BMI; kg/m2) was calculated, then categorized according to the World Health Organization’s classification for adults; underweight (<18.5), normal weight (18.5-24.9), overweight/obese ≥25.0.^28^

***Smoking:*** This was assessed by asking participants how often they smoke tobacco (cigarettes only), with the response alternatives; ≥20/day, 11-19/day, 1-10/day, Occasionally, Never. The first 4 responses were merged as “Smokers” (versus Non-smokers).

***Self-rated health:*** This was assessed with the question—“I perceive my physical and mental health as….” With 5 response alternatives: Very poor, Poor, Neither good nor bad, Good, Very good. These were grouped as: Very poor/Poor, Neither good nor bad, and Good/Very good.

***Age and sex:*** These were self-reported and included as continuous and categorical variables, respectively. Analyses were stratified based (approximately) on the mean age; that is, 18-39 years, and 40-65 years.

### Data Analysis

**Figure 1.**
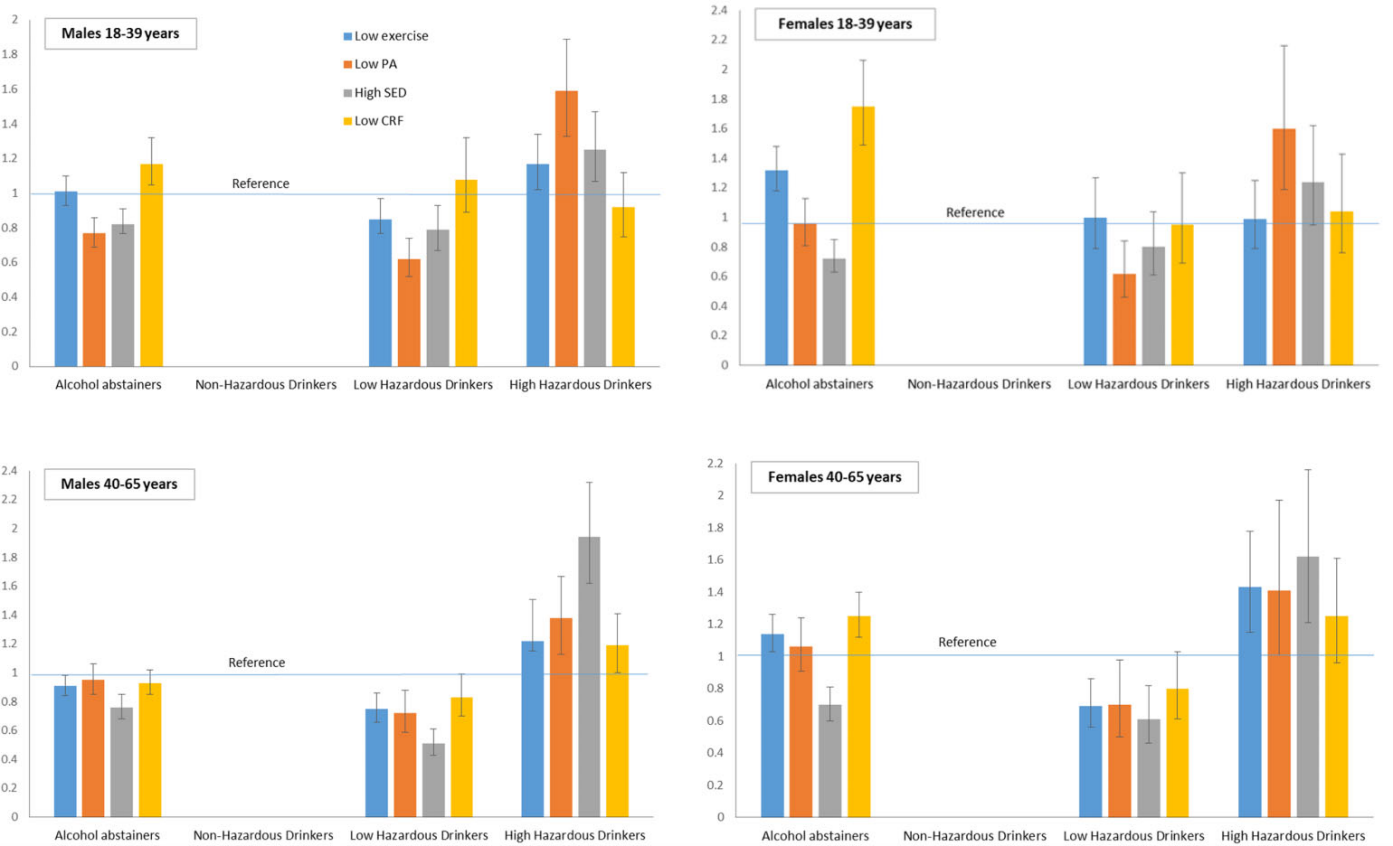
Results of binary logistic regression analyses showing the odds of engaging in: low exercise, low physical activity (PA), high sedentary behavior (SED), and of having low cardiorespiratory fitness (CRF). all models adjusted for body mass index (BMI). drinking thresholds (AUDIT-C): non-hazardous = 1-3 (women), 1-4 (men); low hazardous = 4-5 (women), 5-6 (men), high hazardous ≥ 6 (women), ≥ 7 (men).

For descriptive data; means and standard deviations (SD) were calculated for continuous variables; total n and percentages (%) for categorical variables. Binary logistic regression was used to estimate the odds of engaging in low levels of habitual physical activity and structured exercise, high levels of leisure time sedentary behavior, and of having low cardiorespiratory fitness. Results are expressed as odds ratios (ORs) with corresponding 95% confidence intervals (CIs) and p-values. As per convention, values <0.05 were considered statistically significant. For reader information, p-values approaching significance (<0.1) are also shown. In all regression models, the reference category was non-hazardous drinking. Crude and adjusted (for body mass index and education) models were calculated (Table 2: males, Table 3: females). Adjusted models only are presented in Figure 1. All analyses were performed using SPSS version 24.

**Table 1.** Participant Characteristics Stratified by Drinking Status (n = 47,559).

	Alcohol abstainers (n = 4,587) n (%)	Non-hazardous drinkers* (n = 26,965) n (%)	Hazardous drinkers (n = 16007)	
			Low* (n = 12,174) n (%)	High* (n = 3,833) n (%)
**Sex**				
Women	2,308 (50.3)	11,066 (41.0)	4,869 (40.0)	1,046 (27.3)
Men	2,279 (49.7)	15,899 (58.9)	7,305 (60.0)	2,787 (71.7)
**Age**; mean (SD)	40.3 (11.8)	42.3 (11.5)	41.2 (12.2)	38.4 (13.4)
**Education (SSYK)**				
Primary school	394 (8.6)	755 (2.8)	268 (2.2)	111 (2.9)
Secondary/ tertiary (2 years)	2,436 (53.1)	12,485 (46.3)	5,807 (47.7)	2,200 (57.4)
Vocational tertiary (3-4 years)	656 (14.3)	5,366 (19.9)	2,508 (20.6)	701 (18.3)
Theoretical tertiary (≥ 3 years)	1,101 (24.0)	8,359 (31.0)	3,591 (29.5)	821 (21.4)
**Relationship status**				
None	627 (13.7)	3,324 (12.2)	1,441 (11.8)	523 (13.6)
Live separately	962 (21.0)	4,796 (17.5)	2,678 (22.0)	1,278 (33.3)
Live together	2,998 (65.4)	19,237 (70.3)	8,057 (66.2)	2,032 (53.1)
**Smoking status**				
Non-smoker	3,943 (86.1)	23,767 (86.9)	9,609 (79.0)	2,501 (65.3)
Smoker	639 (14.0)	3,570 (13.1)	2,559 (21.0)	1,331 (34.7)
**Body Mass Index**				
Underweight	61 (1.4)	278 (1.1)	104 (0.9)	34 (1.0)
Normal weight	1,683 (39.6)	11,406 (44.6)	4,863 (42.6)	1,367 (38.6)
Overweight/obese	2,504 (59.0)	13,879 (54.3)	6,444 (56.5)	2,143 (60.5)
**Self-rated health**				
Very poor/Poor	505 (11.3)	2,011 (7.4)	846 (7.0)	417 (10.9)
Neither good nor bad	1,245 (27.2)	7,366 (27.0)	3,390 (27.9)	1,236 (32.3)
Good/Very good	2,829 (61.8)	17,959 (65.7)	7,933 (65.2)	2,178 (56.9)
**Exercise frequency**				
Low (never/sometimes)	1900 (41.7)	8579 (31.9)	3658 (31.1)	1382 (36.2)
≥ Once per week	2659 (58.3)	18321 (68.1)	8491 (69.9)	2442 (63.8)
**Habitual physical activity**				
Low (never/once per week)	721 (19.2)	3051 (13.9)	1374 (13.9)	651 (21.1)
≥ Several times/week	3039 (80.8)	18893 (86.1)	8513 (86.1)	2434 (78.9)
**Sedentary behavior**				
High (≥75% of the time)	745 (16.3)	3053 (11.3)	1569 (12.9)	755 (19.7)
<75% of the time	3838 (83.7)	23885 (88.7)	10599 (87.1)	3075 (80.3)
**Cardiorespiratory fitness**				
Low (≤32 ml/min/kg)	1445 (47.9)	7599 (38.3)	3195 (35.3)	1024 (36.7)
>32 ml/min/kg	1566 (52.1)	12246 (61.7)	5856 (64.7)	1763 (63.3)

* Drinking thresholds based on AUDIT-C points: Abstainers = 0 (all); Non-hazardous drinkers = 1-3 (women), 1-4 (men); Low hazardous drinkers = 4-5 (women), 5-6 (men), High hazardous drinkers ≥ 6 (women), ≥ 7 (men).

**Table 2.** Logistic Regression Models Showing the Odds of Engaging in Low Exercise, Low Physical Activity, High Sedentary Behavior, and of Having Low CRF.

Males (n = 28,270)	18-39 years (n = 12,860)				40-65 years (n = 15,410)			
	Crude		Adjusted		Crude		Adjusted	
	OR	95% CI	OR	95% CI	OR	95% CI	OR	95% CI
**Low Exercise** **(Never/Sometimes)**								
Non-Hazardous (Ref)								
Abstainers	0.99	0.91-1.07	1.01	0.93-1.10	0.86	**0.80-0.93’’’**	0.91	**0.84-0.98’**
Low-Hazardous	0.87	**0.76-0.99’**	0.85	**0.77-0.97’**	0.69	**0.61-0.79’’’**	0.75	**0.66-0.86’’’**
High-Hazardous	1.14	**1.00-1.30’**	1.17	**1.02-1.34’**	1.43	**1.26-1.63’’’**	1.22	**1.15-1.51’’’**
**Low Physical Activity** **(Never/Once per week)**								
Non-Hazardous (Ref)								
Abstainers	0.74	**0.67-0.83’’’**	0.77	**0.69-0.86’’’**	0.88	**0.79-0.98’**	0.95	0.85-1.06
Low-Hazardous	0.65	**0.55-0.76’’’**	0.62	**0.52-0.74’’’**	0.65	**0.54-0.77’’’**	0.72	**0.59-0.88’’**
High-Hazardous	1.53	**1.30-1.80’’’**	1.59	**1.33-1.89’’’**	1.53	**1.28-1.84’’’**	1.38	**1.13-1.67’’**
**Low Cardiorespiratory Fitness** **(<32 ml/min/kg)**								
Non-Hazardous (Ref)								
Abstainers	1.09	0.99-1.21	1.17	**1.05-1.32’’**	0.82	**0.75-0.89’’’**	0.93	0.85-1.02
Low-Hazardous	1.00	0.85-1.19	1.08	0.89-1.32	0.77	**0.66-0.90’’**	0.83	**0.70-0.99’**
High-Hazardous	0.99	0.83-1.17	0.92	0.75-1.12	1.29	**1.10-1.51’’**	1.19	**1.00-1.41’**
**High Sedentary Behavior** **(≥75% of leisure time)**								
Non-Hazardous (Ref)								
Abstainers	0.82	**0.74-0.91’’’**	0.82	**0.77-0.91’’’**	0.74	**0.67-0.83’’’**	0.76	**0.68-0.85’’’**
Low-Hazardous	0.77	**0.66-0.89’’**	0.79	**0.67-0.93’’**	0.47	**0.40-0.56’’’**	0.51	**0.43-0.61’’’**
High-Hazardous	1.29	**1.14-1.51’’**	1.25	**1.07-1.47’’**	2.09	**1.76-2.48’’’**	1.94	**1.62-2.32’’’**

*Note*. Boldface indicates statistical significance (‘p < 0.05, ‘‘p < 0.01, ‘‘‘p < 0.001).

**Table 3.** Logistic Regression Models Showing the Odds of Engaging in Low Exercise, Low Physical Activity, High Sedentary Behavior, and of Having Low CRF.

Females (n = 19,289)	18-39 years (n = 8,486)				40-65 years (n = 10,813)			
	Crude		Adjusted		Crude		Adjusted	
	OR	95% CI	OR	95% CI	OR	95% CI	OR	95% CI
**Low Exercise (Never/Sometimes)**								
Non-Hazardous (Ref)								
Abstainers	1.30	**1.16-1.45’’’**	1.32	**1.18-1.48’’’**	1.16	**1.06-1.28’’**	1.14	**1.03-1.26’**
Low-Hazardous	0.96	0.77-1.19	1.00	0.79-1.27	0.63	**0.51-0.78’’’**	0.69	**0.56-0.86’’**
High-Hazardous	1.03	0.83-1.29	0.99	0.79-1.25	1.57	**1.28-1.93’’’**	1.43	**1.15-1.78’’**
**Low Physical Activity** **(Never/Once per week)**								
Non-Hazardous (Ref)								
Abstainers	0.99	0.84-1.16	0.96	0.81-1.13	1.11	0.96-1.29	1.06	0.91-1.24
Low-Hazardous	0.62	**0.47-0.82’’**	0.62	**0.46-0.84’’**	0.65	**0.47-0.89’’**	0.70	**0.50-0.98’**
High-Hazardous	1.60	**1.20-2.12’’**	1.60	**1.19-2.16’’**	1.53	**1.12-2.08’’**	1.41	**1.01-1.97’**
**Low Cardiorespiratory Fitness** **(<32 ml/min/kg)**								
Non-Hazardous (Ref)								
Abstainers	1.56	**1.36-1.79’’’**	1.75	**1.49-2.06’’’**	1.23	**1.12-1.36’’’**	1.25	**1.12-1.40’’’**
Low-Hazardous	0.79	0.60-1.04	0.95	0.69-1.30	0.71	**0.56-0.90’’**	0.80	0.61-1.03
High-Hazardous	1.26	0.96-1.65	1.04	0.76-1.43	1.39	**1.12-1.75’’**	1.25	0.96-1.61
**High Sedentary Behavior (≥75% of leisure time)**								
Non-Hazardous (Ref)								
Abstainers	0.72	**0.62-0.83’’’**	0.72	**0.63-0.85’’’**	0.76	**0.66-0.87’’’**	0.70	**0.60-0.81’’’**
Low-Hazardous	0.73	**0.57-0.94’**	0.80	0.61-1.04	0.56	**0.43-0.74’’’**	0.61	**0.46-0.82’’**
High-Hazardous	1.36	**1.06-1.74’**	1.24	0.95-1.62	1.76	**1.34-2.30’’’**	1.62	**1.21-2.16’’**

*Note*. Boldface indicates statistical significance (‘p < 0.05, ‘‘p < 0.01, ‘‘‘p < 0.001).

## Results

### Participant Characteristics

Participant characteristics are show in Table 1. Of the total sample (n = 47,559) 59.4% were males (mean age = 41.5 years; SD = 11.9); 9.7% were alcohol abstainers, 56.9% were non-hazardous drinkers, and 33.4% were hazardous drinkers (low = 25.4%, high = 8.0%). Mean AUDIT-C scores were as follows: total sample = 3.3 (SD = 2.0); males = 3.8 (SD = 2.1), females = 2.7 (SD = 1.7). All study participants were employed, 29% had a tertiary/university degree, 83.1% were non-smokers, 87.3% were in a relationship (20.2% living apart), 37.7% were overweight, and 64.5% rated their health as good or very good (versus 7.9% poor/very poor). Compared to all drinking categories, alcohol abstainers reported the highest proportion of poor/very poor health, the lowest level of education, and the highest average BMI. Compared to non-hazardous drinkers, high-hazardous drinkers (women and men scoring ≥6 and ≥7 AUDIT-C points, respectively) had less tertiary education, and were more likely to be: single, smokers, overweight, and to report poor/very poor health.

### Physical Activity Habits and Cardiorespiratory Fitness Among Males

Figure 1 shows the results of adjusted binary logistic regression analyses (non-hazardous drinkers as reference), and the odds of engaging in low exercise and physical activity, high sedentary behavior, and of having low CRF. Analyses are stratified by age and gender.

**Among younger males (18-39 years),** compared to non-hazardous drinkers, high-hazardous drinkers did less structured exercise (OR = 1.17, CI = 1.02-1.34, p = .019), less habitual physical activity (OR = 1.59, CI = 1.33-1.89, p = .000), and were more sedentary in their leisure time (OR = 1.25, CI = 1.07-1.47, p = .005). Conversely, compared to non-hazardous drinkers, low-hazardous drinkers engaged in more exercise (OR = 0.85, CI = 0.77-0.97, p = .019) and physical activity (OR = 0.62, CI = 0.52-0.74, p = .000), and were less sedentary (OR = 0.79, CI = 0.67-0.93, p = .005). Compared to non-hazardous drinkers, alcohol abstainers were less sedentary (OR = 0.82, CI = 0.77-0.91, p = .000) and more physically active (OR = 0.77, CI = 0.69-0.86, p = .000), but had lower CRF (OR = 1.17, CI = 1.05-1.32, p = .005).

**Among older males (40-65 years)**, compared to non-hazardous drinkers, high-hazardous drinkers did less structured exercise (OR = 1.22, CI = 1.15-1.51, p = .000), less habitual physical activity (OR = 1.38, CI = 1.13-1.67, p = .001), spent higher durations of their leisure time sedentary (OR = 1.94, CI = 1.62-2.32, p = .000), and had lower CRF (OR = 1.19, CI = 1.00-1.41, p = .044). Compared to non-hazardous drinkers, low-hazardous drinkers did more exercise (OR = 0.75, CI = 0.66-0.86, p = .000) and physical activity (OR = 0.72, CI = 0.59-0.88, p = .001), were less sedentary (OR = 0.51, CI = 0.43-0.61, p = .000), and had higher CRF (OR = 0.83, CI = 0.70-0.99, p = .044). Compared to non-hazardous drinkers, alcohol abstainers were less sedentary (OR = 0.76, CI = 0.68-0.85, p = .000), and did more exercise (OR = 0.91, CI = 0.84-0.98, p = .013). In crude models only, abstainers also had higher CRF (OR = 0.82, CI = 0.75-0.89, p = .000).

### Physical Activity Habits and Cardiorespiratory Fitness Among Females

**Among younger females (18-39 years)**, compared to non-hazardous drinkers, high-hazardous drinkers did less habitual physical activity (OR = 1.60, CI = 1.19-2.16, p = .002). In crude models only, they were also more sedentary (OR = 1.36, CI = 1.06-1.74, p = .014). Compared to non-hazardous drinkers, low-hazardous drinkers engaged in more physical activity (OR = 0.62, CI = 0.46-0.84, p = .002). In crude models only, they were also less sedentary (OR = 0.73, CI = 0.57-0.94, p = .014). Compared to non-hazardous drinkers, alcohol abstainers did less exercise (OR = 1.32, CI = 1.18-1.48, p = .000), had lower CRF (OR = 1.75, CI = 1.49-2.06, p = .000), but were less sedentary (OR = 0.72, CI = 0.63-0.85, p = .000).

**Among older females (40-65 years)**, compared to non-hazardous drinkers, high-hazardous drinkers did less structured exercise (OR = 1.43, CI = 1.15-1.78, p = .001), less habitual physical activity (OR = 1.41, CI = 1.01-1.97, p = .040), were more sedentary (OR = 1.62, CI = 1.21-2.16, p = .001), and tended to have lower CRF (adjusted OR = 1.25, CI = 0.96-1.61, p = .091; Crude OR = 1.39, CI = 1.12-1.75, p = .004). Compared to non-hazardous drinkers, low-hazardous drinkers engaged in more exercise (OR = 0.69, CI = 0.56-0.86, p = .001) and physical activity (OR = 0.70, CI = 0.50-0.98, p = .040), and were less sedentary (OR = 0.70, CI = 0.60-0.81, p = .001). A trend toward higher CRF was also observed (adjusted OR = 0.80, CI = 0.61-1.03, p = .091; crude OR = 0.71, CI = 0.56-0.90, p = .004). Compared to non-hazardous drinkers, alcohol abstainers did less exercise (OR = 1.14, CI = 1.03-1.26, p = .010), and had lower CRF (OR = 1.25, CI = 1.12-1.40, p = .000), but were less sedentary (OR = 0.70, CI = 0.60-0.81, p = .000).

## Discussion

To our knowledge, this is the first general population study to examine the full spectrum of physical activity and cardiorespiratory fitness (CRF) in adults based on alcohol consumption status. Notable differences were observed between the alcohol consumption groups. With the partial exception of young females, the heaviest (“high-hazardous”) drinkers engaged in less structured exercise and were less physically active compared to non-hazardous drinkers. These results are consistent with recent studies showing that adults receiving treatment for AUD are physically inactive, compared to age-gender matched controls.^4,5^ Across all groups, high-hazardous drinkers were also more sedentary (sitting/reclining) in their leisure time than non-hazardous drinkers. These differences were more pronounced among older (40-65 years) adults, especially males, where the proportion that was highly sedentary (≥75% of the time) during leisure (20.1%) was approximately twice that of highly sedentary non-hazardous drinkers (10.1%) (data not shown). Compared to non-hazardous drinkers, older males in the heaviest drinking category were the least active group overall, with higher odds of low activity levels across all 4 measures. Predictably, mean CRF scores were higher among younger adults, who also exercised more frequently than older adults.

Activity patterns among alcohol abstainers were mixed. A consistent finding was that, compared to non-hazardous drinkers, abstainers were less sedentary in their leisure time. With the exception of older males, abstainers also had lower CRF levels. Previous research has shown that abstainers are a diverse group consisting of both healthy individuals and those with health problems limiting their ability to exercise.^29,30^ Abstainers in the current study had the lowest self-rated health of all participants. However, with the exception of young females, abstainers were also over-represented in the highest (≥5 times/week) exercise frequency category (data not shown), illustrating the heterogeneity of this group.

Several population-based studies have shown a positive association between alcohol consumption and physical activity. In a US study (n = 230,856), compared to abstainers, light, moderate, and heavy drinkers exercised 5.7, 10.1, and 19.9 additional minutes per week.^31^ Moreover, drinking was associated with a 10.1 percentage point increase in the probability of exercising vigorously. Few studies have explored moderators of the relationship between alcohol use and physical activity. Consistent with our finsings, however, one investigation found that gender and age are important factors. Lisha and colleagues^32^ assessed survey responses from over 30,000 adults in the US, and found a positive association between vigorous exercise and alcohol use that was strongest in those aged 50 years or less. They also found that the association between moderate exercise and alcohol use was strongest in men.^32^ A recent Brazilian study involving a nationally representative sample of adults (n = 60,202) found that weekly alcohol consumption was associated with a higher level of physical activity among young, middle aged and older adults.^33^ Similar to our findings, heavy drinking (defined as “almost daily alcohol consumption”) was associated with lower physical activity among middle-aged adults, but higher levels among young women. The “heavy drinking exerciser” phenomenon has been described previously. Leasure and colleagues postulate a psychobiological explanation, noting that the rewarding neural effects of both exercise and alcohol consumption provide a useful framework for understanding these associations.^34^ They also describe how social pressures to “work hard and play hard” may increase drinking and exercise frequency, especially among younger adults.

Unlike several investigations,^31,33,35^ we did not observe a positive association between alcohol consumption and habitual physical activity or exercise frequency. Indeed, the opposite trend was found; the heaviest drinkers were *less* physically active than non-hazardous drinkers. This inconsistency could be attributable to differences in methodology. Previous studies have often used abstainers as their reference category,^29,31^ whereas we used non-hazardous drinkers. Considering the health issues reported by abstainers,^30^ this may not be the optimal reference group for assessing these relationships. Non-hazardous drinkers report fewer health problems and are more representative of drinking behavior in the general population.^29,30^ Another key difference is that we divided hazardous drinkers into 2 groups (low and high), based on the distribution of AUDIT-C scores. This categorization revealed associations that may not have been detected in studies of “risky” or “heavy” drinkers, where the consumption categories included a wider distribution of drinkers compared to the low/high hazardous categories used here. Our findings indicate that hazardous drinkers are a heterogeneous group, with significant differences in physical activity habits between those scoring at the “low” versus “high” end of this category.

The current findings have potential implications for the prevention and treatment of alcohol-related problems. First, they suggest that middle-aged adults, whose alcohol consumption falls within the high-hazardous level identified here, could be appropriate targets for physical activity interventions. Second, as habitual physical activity (e.g. walking) was lower, and sedentary behavior (sitting/reclining) was consistently higher among the heaviest drinkers, interventions should promote not only structured exercise, but also target reductions in leisure-time sedentary behavior; for example, by replacing extended periods of sitting with standing or short walks. Moreover, physical activity assessments should ideally include questions about sedentary behavior, as some drinkers may exercise regularly but remain sedentary outside these bouts of structured physical activity. Extended durations of sedentary behavior, combined with the higher than average tobacco use reported by these participants, increases their risk for cardiometabolic disease.^36^ Third, although hazardous drinkers are a diverse group in terms of their exercise habits, we found that those presenting with AUDIT-C scores ≥6 points (females) and ≥7points (males) engaged in less physical activity than hazardous drinkers scoring below these cut-points. Thus, sex-specific AUDIT-C scores could be useful indicators for identifying adults who drink too much *and* move too little.

Strengths of the study include the large participant sample, the objective measurement of CRF and questions assessing exercise, physical activity and leisure-time sedentary behavior. Previous studies have mostly been limited to one measure of physical activity—often “exercise.”^35^ Our sample is unique in that all participants were employed, so the findings are likely generalizable to populations of employed adults in other high-income countries. Some potential limitations are acknowledged. The physical activity items were self-reported, which may lead to overestimation of activity levels. These questions have not been validated using objective measures or larger validated questionnaires, respectively. However, related studies show that single-item measures of physical activity are robust predictors of health outcomes,^37^ and previous studies using HPI data have demonstrated the predictive validity of these single-item questions.^38^ Alcohol consumption was assessed using AUDIT-C (first 3 items). Studies using the entire AUDIT questionnaire could potentially identify relationships between harmful drinking, dependence, and physical activity levels. Lastly, we could not determine how many participants were above or below recommended physical activity levels,^39^ nor could we assess differences in exercise intensity per se. Studies measuring these factors could help to identify the proportion of hazardous drinkers that do not meet physical activity guidelines.

## Conclusion

Our findings highlight the complex relationship between physical activity and alcohol use, which appears to be moderated by sex and age. Middle-aged adults, particularly males, who drink at the “high-hazardous” levels identified here, could be appropriate targets for physical activity interventions. Our results also suggest that treatment interventions for hazardous drinking should aim to increase structured exercise, while also reducing leisure-time sedentary behavior. As the associations between alcohol consumption and physical activity were less consistent among younger adults, more research is needed focusing on the drinking and exercise habits of younger populations.

**SO WHAT?**

**What is Already known on this Topic?**

Research has shown a positive association between alcohol consumption and physical activity levels in adults. Studies measuring sedentary behavior (too much sitting) are lacking.

**What does this Article Add?**

Compared to non-hazardous drinkers, high-hazardous drinkers were *less* physically active and more sedentary. Hazardous drinkers are a heterogeneous group with respect to their physical activity and exercise behaviors.

**What are the Implications for Health Promotion Practice or Research?**

Health promotion strategies should encourage high-hazardous drinkers to increase their habitual physical activity levels and minimize sedentary behavior.
